# Phytoseiid mites from tropical fruit trees in Bahia State, Brazil (Acari, Phytoseiidae)

**DOI:** 10.3897/zookeys.533.5981

**Published:** 2015-10-09

**Authors:** Izabel Vieira de Souza, Poliane Sá Argolo, Manoel Guedes Correa Gondim Júnior, Gilberto José de Moraes, Maria Aparecida Leão Bittencourt, Anibal Ramadan Oliveira

**Affiliations:** 1 Universidade Estadual de Santa Cruz, Rodovia Ilhéus – Itabuna, km 16, 45.662-900, Ilhéus, BA, Brazil; 2 Universidade Federal Rural de Pernambuco, Área de Fitossanidade, 52171-900, Recife, PE, Brazil; 3 CNPq Researcher; Departamento de Entomologia e Acarologia, Escola Superior de Agricultura “Luiz de Queiroz”, Universidade de São Paulo, 13418-900, Piracicaba, SP, Brazil

**Keywords:** Acarology, Predatory mites, Taxonomy, Biological Control

## Abstract

The cultivation of tropical fruit trees has grown considerably in the state of Bahia, northeastern Brazil. Some of these have been severely attacked by phytophagous mites, which are usually controlled by the use of chemical pesticides. However, there is today a growing interest for the adoption of less aggressive measures of pest control, as for example the use of predatory mites. Most of the plant-inhabiting predatory mites belong to the family Phytoseiidae. The objective of this paper is to report the phytoseiid species found in an intensive survey conducted on cultivated tropical fruit trees in fifteen localities of the southern coast of Bahia. Measurements of relevant morphological characters are provided for each species, to complement the understanding of the morphological variation of these species. Twenty-nine species of sixteen genera were identified. A key was elaborated to assist in the separation of these species. Fifteen species are reported for the first time in the state, raising to sixty-six the number of species of this family now known from Bahia. Seventy-two percent of the species collected belong to Amblyseiinae, followed by Typhlodrominae (21%) and Phytoseiinae (7%). The most diverse genus was *Amblyseius*. *Amblyseius operculatus* De Leon was the most frequent and abundant species. Studies should be conducted to evaluate the possible role of the most common predators as control agents of the phytophagous mites co-occurring with them.

## Introduction

Cultivation of tropical fruit trees has grown considerably in the state of Bahia, northeastern Brazil, in the last years (Santos-Serejo et al. 2009). Several mite species have been reported on those plants, some causing economic losses (Moraes and Flechtmann 2008). These are usually controlled by the use of chemical pesticides.

However, there is today a growing interest on the use of less aggressive and less toxic strategies to control those organisms. Predatory mites of the family Phytoseiidae are considered important biological control agents of pest mites, and some phytoseiids are commercially available for the control of pest mites in several countries (Hoy 2011). There is an interest to implement the use of phytoseiids for the biological control of pest mites in orchards of tropical fruit trees in coastal Bahia, and the determination of the naturally occurring phytoseiids in that area is considered the first step in the implementation of a biological control program.

The objective of this paper is to report the phytoseiid species found in an intensive survey conducted on cultivated tropical fruit trees in the southern coastal region of Bahia, providing a key to help the separation of the species collected.

## Materials and methods

**Figure 1.**
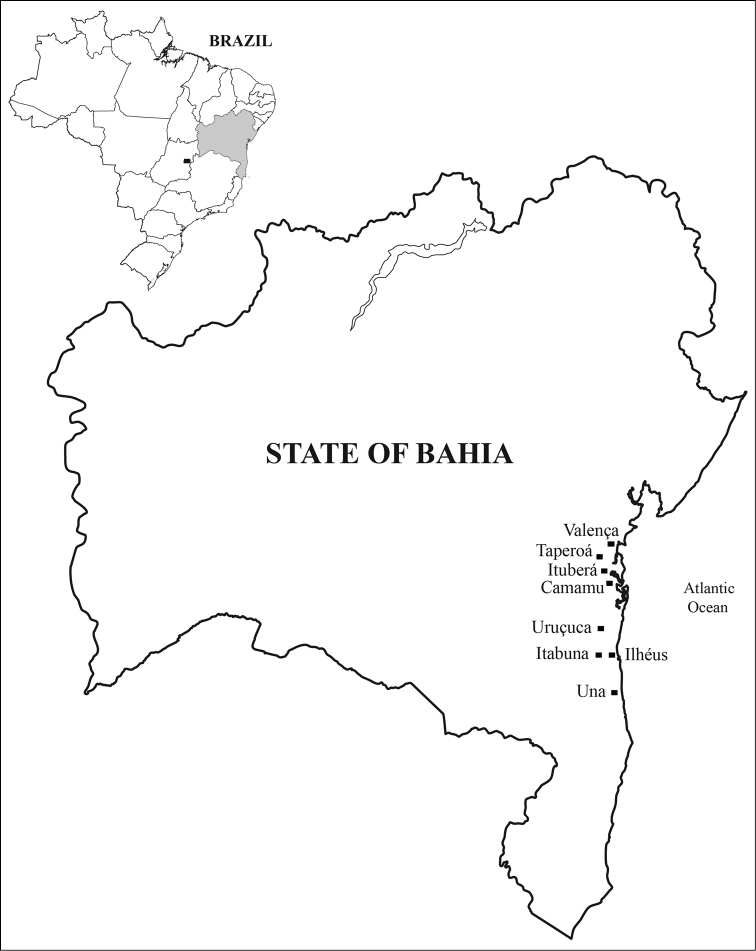
Map locating the municipalities in the State of Bahia, Brazil, where collections were conducted.

Samples were collected from March 2007 to January 2010 in fifteen localities of eight municipalities (Figure 1, Table 1). These consisted mainly of leaves and, when present, flowers and fruits of 21 species of tropical fruit trees (Table 2).

**Table 1.** Localities in the State of Bahia, Brazil, from which phytoseiid mites were collected between March 2007 and January 2010.

Municipality	Sampling locality	Coordinates
Camamu	Fazenda Bela Vista	13°58'35"S, 39°09'23"W
Ilhéus	UESC	14°47'53"S, 39°10'20"W
	Fazenda Terra Nova	14°43'52"S, 39°09'16"W
	Sítio Agrotropical	14°47'00"S, 39°14'42"W
Itabuna	Fazenda Monte Alegre	14°43'29"S, 39°20'42"W
Ituberá	Colônia de Japoneses	13°46'29"S, 39°11'04"W
	Fazenda Frupical	13°45'23"S, 39°10'58"W
	Fazenda Kamuí	13°45'11"S, 39°10'50"W
Taperoá	Fazenda Nossa Senhora Auxiliadora	13°33'52"S, 39°12'06"W
Una	Estação Lemos Maia-CEPLAC	15°21'18"S, 38°59'56"W
Uruçuca	Fazenda Liberdade	14°35'52"S, 39°19'55"W
Valença	Fazenda Barra	13°21'05"S, 39°19'57"W
	Fazenda Formiga	13°18'01"S, 39°15'02"W
	Sítio Sabino	13°18'59"S, 39°15'46"W
	Sítio São Jorge	13°17'23"S, 39°18'05"W

**Table 2.** Species of tropical fruit trees from which phytoseiid mites were collected in the State of Bahia, Brazil, between March 2007 and January 2010.

Family	Plant species
Anacardiaceae	*Anacardium occidentale* L.
	*Mangifera indica* L.
	*Spondias mombin* L.
Annonaceae	*Annona muricata* L.
	*Annona squamosa* L.
Arecaceae	*Cocos nucifera* L.
	*Elaeis guineensis* Jacq.
	*Euterpe oleracea* Mart.
Caricaceae	*Carica papaya* L.
Ebenaceae	*Diospyros kaki* L.
Lauraceae	*Persea americana* L.
Malpighiaceae	*Malpighia emarginata* D.C.
Moraceae	*Artocarpus integrifólia* Lam.
Musaceae	*Musa sapientum* L.
Myrtaceae	*Psidium guajava* L.
	*Syzygium malaccense* (L.) Merr. & L.M. Perry
Passifloraceae	*Passiflora edulis* Sims.
Rubiaceae	*Genipa americana* L.
Sapotaceae	*Pouteria caimito* (Ruiz & Pav.) Radlk.
Sterculiaceae	*Theobroma cacao* L.
	*Theobroma grandiflorum* Schum.

Phytoseiid mites were mounted in Hoyer’s medium, identified and measured under a phase-contrast microscope (Motic® B3 Professional Series). Under each species mentioned in the Results section, information concerning the specimens examined is given in the following order: sampling locality, plant species, month and year of the collection, number and sex of specimens. Measurements are given in micrometers, corresponding to the average for the structures measured followed in parentheses by the respective ranges. Numbers of teeth on the fixed and movable cheliceral digits do not include the respective apical teeth. Setae not referred to in the Results section should be considered absent.

Idiosomal setal notation adopted is that of Lindquist and Evans (1965), as applied to phytoseiids by Rowell et al. (1978) and Chant and Yoshida-Shaul (1989) for the dorsal surface, and by Chant and Yoshida-Shaul (1991) for the ventral surface. Macrosetal notation is that of Muma et al. (1970). The system of classification follows that of Chant and McMurtry (2007). The name adopted for each species is that mentioned in the Phytoseiidae Database (Demite et al. 2014), followed by the names attributed to the species in the original description, in the catalog of Moraes et al. (2004) and in the comprehensive work of Chant and McMurtry (2007).

Voucher specimens were deposited in the mite reference collection of Universidade Estadual de Santa Cruz (UESC), Ilhéus, Bahia.

## Results

In total, 564 phytoseiid specimens were collected, belonging to twenty-nine species of sixteen genera, as subsequently reported. Most specimens were collected from *Cocos nucifera* (41%), followed by *Theobroma cacao* (24%), and *Psidium guajava* (16%) (Tables 3 and 4).

**Table 3.** Numbers of specimens and of species of phytoseiid mites collected on each sampled plant species in the State of Bahia, Brazil, between March 2007 and January 2010.

Family	Plant species	Nr. *	Specimens	Species
				
Anacardiaceae	*Anacardium occidentale*	1	3	3
	*Mangifera indica*	2	15	6
	*Spondias mombin*	3	1	1
Annonaceae	*Annona muricata*	4	12	4
	*Annona squamosa*	5	2	1
Arecaceae	*Cocos nucifera*	6	234	17
	*Elaeis guineensis*	7	7	5
	*Euterpe oleracea*	8	6	6
Caricaceae	*Carica papaya*	9	9	4
Ebenaceae	*Diospyros kaki*	10	3	3
Lauraceae	*Persea americana*	11	2	1
Malpighiaceae	*Malpighia emarginata*	12	1	1
Moraceae	*Artocarpus integrifolia*	13	6	3
Musaceae	*Musa sapientum*	14	9	8
Myrtaceae	*Psidium guajava*	15	92	13
	*Syzygium malaccense*	16	1	1
Passifloraceae	*Passiflora edulis*	17	5	2
Rubiaceae	*Genipa americana*	18	3	1
Sapotaceae	*Pouteria caimito*	19	2	1
Sterculiaceae	*Theobroma cacao*	20	137	15
	*Theobroma grandiflorum*	21	14	6

* Corresponding to plant species numbers given in Table 4.

**Table 4.** Numbers of phytoseiid mites and diversity of host plants on which each species was collected in the State of Bahia, Brazil, between March 2007 and January 2010.

Phytoseiid species	Nr. of specimens	Plant species nr.*
**Amblyseiinae**		
*Amblyseius operculatus*	133	1, 2, 4, 6, 7, 10, 11, 12, 13, 14, 15, 20, 21
*Amblyseius perditus*	57	2, 6, 7, 13, 14, 15, 20, 21
*Iphiseiodes metapodalis*	53	2, 4, 5, 6, 7, 8, 14, 15, 20, 21
*Iphiseiodes zuluagai*	47	1, 2, 4, 6, 7, 8, 9, 19, 20, 21
*Amblyseius aerialis*	32	6, 9, 15, 20, 21
*Amblyseius tamatavensis*	32	6, 8, 14, 17
*Amblyseius igarassuensis*	28	6, 15, 20
*Typhlodromips theobromae*	15	2, 20
*Amblyseius impeltatus*	10	15
*Iphiseiodes setillus*	8	8, 20
*Paraamblyseius multicircularis*	8	8, 20
*Amblydromalus manihoti*	7	9, 20, 21
*Typhlodromalus peregrinus*	7	6, 9, 15, 20
*Proprioseiopsis neotropicus*	6	6, 10, 14, 20
*Proprioseiopsis dominigos*	4	6, 14, 15
*Arrenoseius urquharti*	3	6, 14
*Proprioseiopsis ovatus*	3	8, 14, 17
*Paraphytoseius orientalis*	2	15
*Phytoscutus sexpilis*	1	15
*Proprioseiopsis pentagonalis*	1	20
*Typhlodromips mangleae*	1	6
**Phytoseiinae**		
*Phytoseius latinus*	21	15
*Phytoseius woodburyi*	12	6, 15
**Typhlodrominae**		
*Leonseius regularis*	56	2, 3, 4, 6, 7, 13, 15, 16, 18, 20
*Cocoseius palmarum*	9	6
*Cocoseius elsalvador*	5	6, 20
*Metaseiulus ferlai*	1	1
*Typhlodromina subtropica*	1	10
*Typhlodromus transvaalensis*	1	6

* Corresponding to plant species numbers given in Table 3.

### Amblyseiinae Muma

#### Amblydromalus manihoti

Taxon classificationAnimaliaMesostigmataPhytoseiidae

(Moraes)

Amblyseius manihoti Moraes et al., 1994: 211.
Typhlodromalus manihoti : Moraes et al. 2004: 200.
Amblydromalus manihoti : Chant and McMurtry 2007: 117.

##### Specimens examined.

UESC, *Carica papaya*, I-2008 (2♀); Fazenda Bela Vista, *Theobroma cacao*, XI-2007 (2♀); Fazenda Frupical, *Theobroma grandiflorum*, VII-2007 (1♀); Fazenda Liberdade, *Carica papaya*, V-2007 (2♀).

##### Female.

Four specimens measured. Dorsal shield 328 (320–338) long, 214 (205–223) wide, *j1* 29 (28–31), *j3* 38 (35–41), *j4* 10 (8–11), *j5* 9 (8–10), *j6* 12 (11–12), *J2* 14 (12–15), *J5* 9 (7–10), *z2* 11 (10–13), *z4* 12 (11–14), *z5* 9 (8–10), *Z1* 14 (13–15), *Z4* 14 (13–17), *Z5* 50 (47–51), *s4* 39 (35–44), *S2* 16 (15–17), *S4* 15 (14–16), *S5* 13 (11–14), *r3* 13 (12–13), *R1* 13 (11–14); distances between *St1*–*St3* 58 (55–60), *St2–St2* 67 (64–73) and *St5–St5* 69 (66–71); ventrianal shield 96 (92–105) long, 59 (56–62) wide at level of *ZV2* and 67 (64–70) wide at level of anus; movable cheliceral digit 32 (31–32) long, with 4 teeth; fixed cheliceral digit 26 (25–26) long, with 11 teeth; calyx of spermatheca 20 (16–23) long; *Sge I* 36 (34–38), *Sge II* 34 (32–37), *Sge III* 38 (36–41), *Sti III* 34 (32–36), *Sge IV* 61 (56–66), *Sti IV* 46 (40–51), *St IV* 80 (75–82).

##### Remarks.

Measurements of the specimens collected are similar to those of the original description. They also agree with those of Moraes et al. (2013), except for the shorter *Z5* [64 (52–70) in the latter].

#### Amblyseius aerialis

Taxon classificationAnimaliaMesostigmataPhytoseiidae

(Muma)

Amblyseiopsis aerialis Muma, 1955: 264.
Amblyseius aerialis : Moraes et al. 2004: 13.
Amblyseius aerialis : Chant and McMurtry 2007: 75.

##### Specimens examined.

UESC, *Theobroma grandiflorum*, IV-2008 (1♀); Fazenda Bela Vista, *Cocos nucifera*, IV-2007 (12♀), *Psidium guajava*, VII-2007 (1♀), *Theobroma cacao*, XI-2007 (1♀); Fazenda Frupical, *Theobroma grandiflorum*, VII-2007 (2♀); Fazenda Liberdade, *Carica papaya*, V-2007 (1♀); Fazenda Nossa Senhora Auxiliadora, *Carica papaya*, VII-2007 (1♀), *Cocos nucifera*, XI-2007 (2♀); Fazenda Terra Nova, *Cocos nucifera*, V-2007 (5♀); Sítio Agrotropical, *Cocos nucifera*, III-2007 (1♂); Sítio São Jorge, *Cocos nucifera*, XI-2007 (1♀, 4♂).

##### Female.

Ten specimens measured. Dorsal shield 386 (364–422) long, 284 (266–309) wide, *j1* 34 (30–37), *j3* 55 (52–59), *j4* 5 (4–8), *j5* 5 (4–6), *j6* 7 (6–8), *J2* 9 (7–11), *J5* 8 (7–8), *z2* 14 (13–16), *z4* 9 (8–13), *z5* 6 (5–7), *Z1* 10 (10–12), *Z4* 133 (120–143), *Z5* 296 (271–315), *s4* 108 (105–111), *S2* 12 (11–14), *S4* 12 (11–14), *S5* 13 (12–13), *r3* 16 (14–17), *R1* 12 (11–13); distances between *St1*–*St3* 65 (63–71), *St2–St2* 83 (79–88) and *St5–St5* 81 (74–87); ventrianal shield 131 (120–142) long, 85 (78–91) wide at level of *ZV2* and 73 (69–77) wide at level of anus; movable cheliceral digit 42 (39–44) long, with 4 teeth; fixed cheliceral digit 34 (33–35) long; calyx of spermatheca 18 (16–20) long; *Sge I* 44 (42–48), *Sge II* 42 (39–45), *Sge III* 61 (55–67), *Sti III* 40 (37–43), *Sge IV* 131 (118–138), *Sti IV* 90 (83–94), *St IV* 80 (75–84).

##### Male.

Five specimens measured. Dorsal shield 309 (294–320) long, 217 (189–248) wide, *j1* 31 (28–32), *j3* 44 (43–45), *j4* 5 (5–6), *j5* 5 (4–6), *j6* 8 (6–9), *J2* 9 (8 10), *J5* 8 (7–8), *z2* 14 (13–15), *z4* 13 (12–13), *z5* 6 (5–7), *Z1* 10 (9–11), *Z4* 103 (102–105), *Z5* 229 (215–241), *s4* 85 (80–90), *S2* 12 (11–14), *S4* 9 (7–11), *S5* 10 (6–12), *r3* 17 (14–20), *R1* 12 (11–13); ventrianal shield 131 (122–137) long and 160 (156–162) wide at anterior corners; shaft of spermatodactyl 19 (16–20) long; *Sge I* 37 (32–39), *Sge II* 35 (30–39), *Sge III* 40 (37–41), *Sti III* 34 (32–35), *Sge IV* 82 (75–90), *Sti IV* 63 (56–70), *St IV* 61 (55–66).

##### Remarks.

The calyx of spermatheca of the specimens collected is about 1.5 times longer than reported by Denmark and Muma (1989) (11–12), but it is comparable to measurements of Brazilian specimens given by Gondim Jr. and Moraes (2001).

#### Amblyseius igarassuensis

Taxon classificationAnimaliaMesostigmataPhytoseiidae

Gondim Jr. & Moraes

Amblyseius igarassuensis Gondim Jr. & Moraes, 2001: 71.
Amblyseius igarassuensis : Moraes et al. 2004: 30.
Amblyseius igarassuensis : Chant and McMurtry 2007: 78.

##### Specimens examined.

UESC, *Psidium guajava*, I-2008 (3♂); Fazenda Bela Vista, *Cocos nucifera*, IX-2007 (2♀, 3♂); Fazenda Monte Alegre, *Theobroma cacao*, V-2007 (3♀), I-2008 (3♀, 1♂), fruit, IX-2007 (1♀); Fazenda Terra Nova, *Theobroma cacao*, IX-2007 (1♀), I-2008 (6♀, 5♂).

##### Female.

Eight specimens measured. Dorsal shield 331 (307–346) long, 216 (197–241) wide, *j1* 25 (23–26), *j3* 37 (35–39), *j4* 8 (6–9), *j5* 7 (6–8), *j6* 10 (10–11), *J2* 10 (9–11), *J5* 9 (8–10), *z2* 11 (9–12), *z4* 11 (11–12), *z5* 7 (6–7), *Z1* 10 (9–11), *Z4* 64 (60–66), *Z5* 141 (130–155), *s4* 59 (56–61), *S2* 11 (10–13), *S4* 10 (10–11), *S5* 10 (9–11), *r3* 12 (11–14), *R1* 10 (9–11); distances between *St1*–*St3* 62 (60–63), *St2–St2* 66 (60–68) and *St5–St5* 62 (59–64); ventrianal shield 110 (102–118) long, 75 (68–79) wide at level of *ZV2* and 65 (62–70) wide at level of anus; movable cheliceral digit 34 (32–35) long, with 3 teeth; fixed cheliceral digit 29 (25–30) long; calyx of spermatheca 10 (9–13) long; *Sge I* 35 (34–38), *Sge II* 34 (33–37), *Sge III* 33 (30–35), *Sti III* 28 (26–29), *Sge IV* 57 (55–60), *Sti IV* 44 (41–46), *St IV* 57 (51–61).

##### Male.

Seven specimens measured. Dorsal shield 252 (243–261) long and 158 (141–174) wide, *j1* 23 (17–28), *j3* 32 (31–34), *j4* 8 (7–9), *j5* 8 (7–8), *j6* 9 (9 10), *J2* 9 (8–10), *J5* 7 (6–8), *z2* 10 (9–12), *z4* 10 (8–11), *z5* 8 (7–8), *Z1* 10 (9–11), *Z4* 48 (41–52), *Z5* 109 (90–138), *s4* 41 (38–44), *S2* 11 (9–12), *S4* 10 (8–11), *S5* 8 (7–10), *r3* 12 (11–13), *R1* 10 (8–11); ventrianal shield 103 (98–110) long and 133 (122–150) wide at anterior corners; shaft of spermatodactyl 18 (17–20) long; *Sge I* 30 (29–34), *Sge II* 28 (26–31), *Sge III* 27 (23–32), *Sti III* 23 (21–27), *Sge IV* 39 (35–47), *Sti IV* 32 (27–38), *St IV* 47 (39–60).

##### Remarks.

Measurements of females of this species are similar to those of the original description, except for the longer *Z5* (91–118 in the original description). This is the first record of this species in Bahia, and the first report of the measurements of males.

#### Amblyseius impeltatus

Taxon classificationAnimaliaMesostigmataPhytoseiidae

Denmark & Muma

Amblyseius impeltatus Denmark & Muma, 1973: 241.
Amblyseius impeltatus : Moraes et al. 2004: 30.
Amblyseius impeltatus : Chant and McMurtry 2007: 78.

##### Specimens examined.

UESC, *Psidium guajava*, VII-2007 (10♀).

##### Female.

Six specimens measured. Dorsal shield 367 (335–410) long, 228 (207–256) wide, *j1* 18 (16–19), *j3* 25 (22–28), *j4* 8 (8–9), *j5* 7 (4–8), *j6* 8 (7–11), *J2* 10 (8–13), *J5* 7 (5–8), *z2* 13 (12–15), *z4* 11 (10–12), *z5* 8 (7–9), *Z1* 12 (10–13), *Z4* 31 (30–33), *Z5* 125 (113–133), *s4* 42 (41–44), *S2* 13 (12–14), *S4* 12 (10–14), *S5* 10 (9–11), *r3* 12 (11–12), *R1* 12 (11–12); distances between *St1*–*St3* 53 (51–54), *St2–St2* 68 (66–69) and *St5–St5* 78 (77–82); ventrianal shield 90 (85–100) long, 80 (74–86) wide at level of *ZV2* and 68 (65–72) wide at level of anus; movable cheliceral digit 30 long, with 3 teeth; fixed cheliceral digit 27 (26–29) long; calyx of spermatheca 6 (5–6) long; *Sge I* 30 (29–30), *Sge II* 32 (31–33), *Sge III* 38 (36–40), *Sti III* 26 (25–27), *Sge IV* 63 (61–65), *Sti IV* 47 (45–48), *St IV* 49 (47–52).

##### Remarks.

Measurements of the specimens collected fit those of the original description. This is the first record of this species in Bahia.

#### Amblyseius operculatus

Taxon classificationAnimaliaMesostigmataPhytoseiidae

De Leon

Amblyseius operculatus De Leon, 1967: 26.
Amblyseius operculatus : Moraes et al. 2004: 45.
Amblyseius operculatus : Chant and McMurtry 2007: 80.

##### Specimens examined.

UESC, *Musa sapientum* VII-2007 (1♀), *Cocos nucifera*, VII-2007 (4♀), I-2008 (3♂), IV-2008 (1♂), *Annona muricata*, VII-2007 (1♀, 1♂), *Elaeis guineensis*, VII-2007 (1♀), *Psidium guajava*, VII-2007 (3♀, 2♂), *Artocarpus integrifolia*, VII-2007 (1♀), *Theobroma grandiflorum*, VIII-2007 (1♀), *Persea americana*, I-2008 (2♀); CEPLAC, *Cocos nucifera*, IV-2008 (1♀), fruits, VI-2008 (1♀), IV-2009 (1♀), V-2009 (1♂), VII-2009 (2♀, 4♂), VIII-2009 (7♀), IX-2009 (2♀, 1♂), X-2009 (5♀, 1♂), XI-2009 (5♀, 1♂), XII-2009 (10♀, 4♂), I-2010 (3♀, 1♂); Colônia de Japoneses, *Cocos nucifera*, XI-2007 (2♀, 4♂); Fazenda Barra, *Anacardium occidentale*, VIII-2007 (1♀), *Theobroma cacao*, XI-2007 (2♀); Fazenda Bela Vista, *Cocos nucifera*, IV-2007 (1♀), IX-2007 (2♀); Fazenda Formiga, *Cocos nucifera*, VIII-2007 (5♀), fruit, VIII-2007 (1♀); Fazenda Frupical, *Musa sapientum*, VII-2007 (1♀), *Theobroma cacao*, VII-2007 (3♀), fruit, VII-2007 (1♀); Fazenda Liberdade, *Theobroma cacao*, V-2007 (1♀); Fazenda Monte Alegre, *Psidium guajava*, V-2007 (1♀), IX-2007 (1♀), V-2008 (1♀), *Mangifera indica*, IX-2007 (1♀), V-2008 (1♀), *Malpighia emarginata*, IX-2007 (1♀), *Cocos nucifera*, I-2008 (1♀, 1♂), V-2008 (1♀, 2♂), *Theobroma cacao*, V-2008 (1♀); Fazenda Nossa Senhora Auxiliadora, *Theobroma cacao*, VII-2007 (1♀), *Cocos nucifera*, VII-2007 (1♀), XI-2007 (1♂), *Psidium guajava*, VII-2007 (1♀), XI-2007 (1♀), *Theobroma grandiflorum*, XI-2007 (5♀); Fazenda Terra Nova, *Theobroma cacao*, V-2007 (2♀), *Diospyros kaki*, V-2007 (1♀), *Cocos nucifera*, IX-2007 (6♀, 1♂); Sítio São Jorge, *Cocos nucifera*, XI-2007 (5♀), XI-2007 (1♀).

##### Female.

Thirteen specimens measured. Dorsal shield 389 (340–428) long, 259 (205–289) wide, *j1* 37 (33–42), *j3* 48 (42–55), *j4* 7 (5–8), *j5* 6 (5–7), *j6* 8 (6–10), *J2* 8 (7–11), *J5* 8 (7–9), *z2* 14 (12–16), *z4* 12 (10–13), *z5* 7 (6–9), *Z1* 10 (8–13), *Z4* 131 (115–148), *Z5* 283 (223–307), *s4* 109 (91–120), *S2* 13 (11–15), *S4* 11 (11–12), *S5* 12 (11–13), *r3* 17 (15–22), *R1* 12 (10–13); distances between *St1*–*St3* 69 (64–71), *St2–St2* 77 (72–80) and *St5–St5* 72 (67–77); ventrianal shield 125 (117–135) long, 77 (70–82) wide at level of *ZV2* and 78 (66–85) wide at level of anus; movable cheliceral digit 45 (42–48) long, with 4 teeth; fixed cheliceral digit 33 (30–36) long; calyx of spermatheca 10 (8–12) long; *Sge I* 48 (45–51), *Sge II* 44 (42–47), *Sge III* 57 (50–63), *Sti III* 41 (37–44), *Sge IV* 121 (105–136), *Sti IV* 81 (65–91), *St IV* 82 (75–89).

##### Male.

Eight specimens measured. Dorsal shield 294 (269–323) long, 217 (197–251) wide, *j1* 29 (27–31), *j3* 44 (42–47), *j4* 6 (5–7), *j5* 5 (5–6), *j6* 8 (7–9), *J2* 9 (7–10), *J5* 7 (6–9), *z2* 13 (10–15), *z4* 11 (10–13), *z5* 7 (6–7), *Z1* 10 (8–11), *Z4* 101 (90–115), *Z5* 218 (208–236), *s4* 82 (77–88), *S2* 12 (11–13), *S4* 11 (10–13), *S5* 12 (10–13), *r3* 15 (14–17), *R1* 11 (10–12); ventrianal shield 129 (115–140) long and 165 (147–181) wide at anterior corners; shaft of spermatodactyl 23 (21–25) long; *Sge I* 38 (35–40), *Sge II* 35 (31–37), *Sge III* 39 (35–43), *Sti III* 31 (27–33), *Sge IV* 81 (72–94), *Sti IV* 60 (55–70), *St IV* 65 (61–70).

##### Remarks.

Measurements of the specimens collected are similar to those of the original description, as well as to those of Gondim Jr. and Moraes (2001).

#### Amblyseius perditus

Taxon classificationAnimaliaMesostigmataPhytoseiidae

Chant & Baker

Amblyseius perditus Chant & Baker, 1965: 16.
Amblyseius perditus : Moraes et al. 2004: 47.
Amblyseius perditus : Chant and McMurtry 2007: 80.

##### Specimens examined.

UESC, *Cocos nucifera*, VII-2007 (1♀), IV-2008 (1♀), *Elaeis guineensis*, VII-2007 (1♀), *Psidium guajava*, VII-2007 (1♀), I-2008 (3♀), *Theobroma grandiflorum*, VII-2007 (1♀), *Artocarpus integrifolia*, I-2008 (2♀); Fazenda Barra, *Theobroma cacao*, XI-2007 (1♀); Fazenda Frupical, *Musa sapientum*, VII-2007 (1♀), *Theobroma cacao*, IV-2007 (1♀), VII-2007 (1♀), IX-2007 (2♀); Fazenda Liberdade, *Cocos nucifera*, V-2007 (5♀), *Mangifera indica*, V-2007 (1♀), *Theobroma cacao*, V-2007 (3♀); Fazenda Monte Alegre, *Psidium guajava*, V-2007 (2♀), IX-2007 (2♀, 1♂), I-2008 (5♀); Fazenda Nossa Senhora Auxiliadora, *Theobroma cacao*, VII-2007 (14♀); Fazenda Terra Nova, *Cocos nucifera*, V-2007 (1♀), IX-2007 (2♀), *Psidium guajava*, I-2008 (5♀).

##### Female.

Fifteen specimens measured. Dorsal shield 345 (333–358) long, 217 (197–238) wide, *j1* 34 (29–36), *j3* 38 (35–43), *j4* 7 (6–8), *j5* 6 (5–7), *j6* 9 (8–10), *J2* 9 (8–11), *J5* 8 (7–9), *z2* 7 (7–8), *z4* 9 (8–10), *z5* 5 (4–7), *Z1* 10 (8–12), *Z4* 59 (54–69), *Z5* 155 (145–174), *s4* 57 (54–67), *S2* 12 (11–13), *S4* 10 (9–11), *S5* 8 (8–10), *r3* 16 (13–17), *R1* 11 (9–12); distances between *St1*–*St3* 60 (57–63), *St2–St2* 68 (65–71) and *St5–St5* 69 (62–74); ventrianal shield 44 (40–49) long, 65 (61–70) wide; anal shield 50 (46–55) long, 67 (64–70) wide; movable cheliceral digit 33 (31–34) long, with 3 teeth; fixed cheliceral digit 26 (25–28) long; calyx of spermatheca 21 (18–28) long; *Sge I* 43 (41–46), *Sge II* 41 (38–45), *Sge III* 45 (40–48), *Sti III* 34 (27–38), *Sge IV* 74 (71–85), *Sti IV* 57 (52–61), *St IV* 65 (62–71).

##### Male.

One specimen measured. Dorsal shield 253 long and 182 wide, *j1* 22, *j3* 31, *j4* 7, *j5* 7, *j6* 8, *J2* 10, *J5* 7, *z2* 8, *z4* 8, *z5* 7, *Z1* 11, *Z5* 50, *s4* 33, *S2* 10, *S4* 9, *S5* 8, *r3* 12, *R1* 8; ventrianal shield 102 long and 166 wide at anterior corners; shaft of spermatodactyl 22 long; *Sge I* 30, *Sge II* 27, *Sge III* 27, *Sti III* 25, *Sge IV* 43, *Sti IV* 32, *St IV* 65.

##### Remarks.

Measurements of the specimens collected fit the redescription given by Denmark and Muma (1989) and those of Gondim Jr. and Moraes (2001). As also reported in those publications, the specimens collected have longer *R1* (5 in the holotype). This is the first record of this species in Bahia.

#### Amblyseius tamatavensis

Taxon classificationAnimaliaMesostigmataPhytoseiidae

Blommers

Amblyseius tamatavensis Blommers, 1974: 144.
Amblyseius tamatavensis : Moraes et al. 2004: 52.
Amblyseius tamatavensis : Chant and McMurtry 2007: 81.

##### Specimens examined.

CEPLAC, *Cocos nucifera*, II-2008 (2♀), fruits, VI-2008 (2♀, 1♂), I-2009 (1♂), II-2009 (2♀), IV-2009 (2♀), VIII-2009 (1♀), X-2009 (1♀), XII-2009 (13♀); Fazenda Frupical, *Musa sapientum*, VII-2007 (1♀); Fazenda Liberdade, *Euterpe oleracea*, V-2007 (1♀); Sítio Agrotropical, *Cocos nucifera*, III-2007 (1♀); Sítio Sabino, *Passiflora edulis*, flowers, XI-2007 (2♀, 2♂).

##### Female.

Seven specimens measured. Dorsal shield 352 (323–379) long and 216 (182–238) wide; *j1* 33 (31–36), *j3* 53 (50–57), *j4* 5 (4–5), *j5* 4 (3–4), *j6* 5 (5–6), *J2* 6 (5–6), *J5* 7 (6–7), *z2* 7 (6–8), *z4* 8 (7–8), *z5* 4 (3–4), *Z1* 6 (6–7), *Z4* 108 (100–115), *Z5* 235 (227–246), *s4* 91 (90–92), *S2* 7 (6–7), *S4* 6 (6–7), *S5* 6 (5–6), *r3* 14 (13–16), *R1* 8 (7–8); distances between *St1*–*St3* 60 (58–63), *St2–St2* 69 (67–72) and *St5–St5* 73 (70–77); ventrianal shield 113 (108–118) long, 95 (89–99) wide at level of *ZV2* and 85 (80–87) wide at level of anus; movable cheliceral digit 37 (36–38) long; fixed cheliceral digit 30 (27–32) long; calyx of spermatheca 17 (16–18) long; *Sge I* 40 (37–42), *Sge II* 39 (35–41), *Sge III* 57 (55–61), *Sti III* 47 (46–47), *Sge IV* 103 (100–105), *Sti IV* 77 (68–80), *St IV* 71 (70–72).

##### Male.

Two specimens measured. Dorsal shield 259, 292 long, 177, 179 wide, *j1* 27, 29, *j3* 43, 45, *j4* 4, 5, *j5* 3, 4, *j6* 5, *J2* 5, *J5* 7, *z2* 6, *z4* 7, *z5* 3, 4, *Z1* 5, 6, *Z4* 82, 90, *Z5* 172, *s4* 67, 69, *S2* 6, 7, *S4* 6 (5–6), *S5* 5, *r3* 12 (11–12), *R1* 7; ventrianal shield 113 (108–118) long and 144 (137–150) wide at anterior corners; shaft of spermatodactyl 17 long; *Sge I* 32 (31–32), *Sge II* 31 (30–31), *Sge III* 38, *Sti III* 34 (33–34), *Sge IV* 67, *Sti IV* 49 (47–50), *St IV* 56 (55–56).

##### Remarks.

Measurements of the specimens collected fit the original description. Moraes et al. (2013) reported shorter *j1*, *j3*, *Sti III*, *Sti IV*, *St IV*, fixed and movable cheliceral digits and the width of ventrianal shield at level of *ZV2* [respectively, 26 (22–30), 42 (40–43), 28 (27–30), 48 (45–50), 51(50–52), 27, 30 and 75] and longer *J2* and *z2* [respectively, 7 (7–8) and 10 (9–10)] in specimens collected in São Paulo state, southeastern Brazil.

#### Arrenoseius urquharti

Taxon classificationAnimaliaMesostigmataPhytoseiidae

Yoshida-Shaul & Chant

Amblyseius urquharti Yoshida-Shaul & Chant, 1988: 2055.
Fundiseius urquharti : Moraes et al. 2004: 89.
Arrenoseius urquharti : Chant and McMurtry 2007: 98.

##### Specimens examined.

Fazenda Monte Alegre, *Musa sapientum*, V-2007 (1♀); Fazenda Nossa Senhora Auxiliadora, *Cocos nucifera*, XI-2007 (1♀, 1♂).

##### Female.

Two specimens measured. Dorsal shield 384 (361–407) long, 378 (366–390) wide, *j1* 17, *j3* 27 (21–32), *j4* 5 (4–5), *j5* 4 (3–5), *j6* 6, *J2* 5 (4–5), *J5* 8 (7–9), *z2* 8 (6–9), *z4* 12 (10–13), *z5* 4 (3–4), *Z1* 4, *Z4* 111 (105–116), *Z5* 104 (95–113), *s4* 86 (79–93), *S2* 12 (11–12), *S4* 8 (7–8), *S5* 7, *r3* 12, *R1* 12; distances between *St1*–*St3* 49 (48–49), *St2–St2* 79 (77–80) and *St5–St5* 128 (124–132); ventrianal shield 143 (140–146) long, 220 (217–223) wide at level of *ZV2* and 142 (133–150) wide at level of anus; movable cheliceral digit 40 long, with 2 teeth; fixed cheliceral digit 32 long; calyx of spermatheca 17 (15–18) long; *St IV* 37 (35–39).

##### Male.

One specimen measured. Dorsal shield 279 long and 212 wide, *j1* 19, *j3* 25 (20–30), *j4* 4, *j5* 4, *j6* 5, *J2* 3, *J5* 9, *z2* 9, *z4* 11, *z5* 3, *Z1* 6, *Z4* 74, *Z5* 59, *s4* 57 (56–58), *S2* 11, *S4* 9, *S5* 10, *r3* 12, *R1* 10; ventrianal shield 136 long and 189 wide at anterior corners; shaft of spermatodactyl 17 long; *St IV* 33.

##### Remarks.

Measurements of the females collected are generally similar to those of the original description, except for the longer *Z4*, *Z5* and *s4* in the females measured in the present work [respectively, 77, 86 and 72 in the original description]. Measurements of the females collected differ from those of Moraes et al. (2013) for the shorter *j3* and the longer width of dorsal shield, *z4*, *Z4*, *S2*, *R1* and calyx of spermatheca [respectively, 33–35, 300–305, 8, 88–92, 98–103, 7–8, 7, 8, 22–23 in the latter]. This is the first record of this genus in Bahia.

#### Iphiseiodes metapodalis

Taxon classificationAnimaliaMesostigmataPhytoseiidae

(El-Banhawy)

Amblyseius metapodalis El-Banhawy, 1984: 132.
Iphiseiodes metapodalis : Moraes et al. 2004: 90.
Iphiseiodes metapodalis : Chant and McMurtry 2007: 98.

##### Specimens examined.

UESC, *Euterpe oleracea*, VII-2007 (1♀), *Cocos nucifera*, VII-2007 (5♀, 1♂), I-2008 (2♀, 1♂), IV-2008 (1♀), *Elaeis guineensis*, VII-2007 (2♀), *Psidium guajava*, VII-2007 (8♀, 1♂); CEPLAC, *Cocos nucifera*, fruit, IV-2009 (1♀); Fazenda Barra, *Annona muricata*, XI-2007 (1♀), *Theobroma cacao*, XI-2007 (1♀); Fazenda Bela Vista, *Cocos nucifera*, IV-2007 (2♀), IX-2007 (1♀, 1♂), XI-2007 (1♀); Fazenda Frupical, *Annona muricata*, XI-2007 (1♀); Fazenda Liberdade, *Theobroma cacao*, V-2007 (2♀), *Mangifera indica*, V-2007 (1♀); Fazenda Monte Alegre, *Musa sapientum*, IX-2007 (1♀), *Cocos nucifera*, IX-2007 (1♀), I-2008 (1♀), V-2008 (2♀), *Annona muricata*, I-2008 (2♀); Fazenda Nossa Senhora Auxiliadora, *Theobroma cacao*, VII-2007 (1♀), *Theobroma grandiflorum*, XI-2007 (1♀); Fazenda Terra Nova, *Cocos nucifera*, V-2007 (1♀), IX-2007 (4♀, 1♂); Sítio Agrotropical, *Cocos nucifera*, III-2007 (2♀); Sítio Sabino, *Annona squamosa* VIII-2007 (2♀).

##### Female.

Eleven specimens measured. Dorsal shield 394 (348–425) long, 313 (282–351) wide, *j1* 23 (20–26), *j3* 34 (32–37), *j4* 5 (4–6), *j5* 5 (4–6), *j6* 6 (5–7), *J2* 7 (6–8), *J5* 8 (7–9), *z2* 6 (5–7), *z4* 6 (5–7), *z5* 5 (4–7), *Z1* 7 (6–7), *Z4* 141 (128–149), *Z5* 192 (184–200), *s4* 149 (140–157), *S2* 7 (6–8), *S4* 6 (5–7), *S5* 6 (5–8), *r3* 6 (6–7), *R1* 7 (6–8); distances between *St1*–*St3* 52 (50–55), *St2–St2* 77 (75–79) and *St5–St5* 103 (95–111); ventrianal shield 121 (111–130) long, 160 (152–165) wide at level of *ZV2* and 115 (110–120) wide at level of anus; movable cheliceral digit 34 (32–36) long, with 4 teeth; fixed cheliceral digit 27 (25–28) long, with 11 teeth; calyx of spermatheca 13 (12–15) long; *Sge I* 63 (58–68), *Sge II* 38 (34–43), *Sge III* 66 (60–71), *Sti III* 31 (29–35), *Sge IV* 138 (130–145), *Sti IV* 82 (76–87), *St IV* 43 (37–47).

##### Male.

Five specimens measured. Dorsal shield 326 (315–340) long, 243 (220–269) wide, *j1* 24 (23–25), *j3* 35 (31–39), *j4* 5 (4–6), *j5* 5 (4–6), *j6* 7 (6–7), *J2* 6 (5–7), *J5* 7 (6–8), *z2* 6 (5–7), *z4* 6 (6–7), *z5* 5 (5–6), *Z1* 6 (5–7), *Z4* 109 (105–118), *Z5* 182 (176–187), *s4* 106 (100–110), *S2* 7 (6–7), *S4* 6 (5–7), *S5* 6 (5–7), *r3* 6 (6–7), *R1* 6 (6–7); ventrianal shield 142 (141–145) long and 191 (186–197) wide at anterior corners; shaft of spermatodactyl 20 (20–22) long; *Sge I* 47 (45–49), *Sge II* 33 (30–35), *Sge III* 44 (42–47), *Sti III* 28 (25–30), *Sge IV* 95 (92–98), *Sti IV* 65 (60–70), *St IV* 44 (42–48).

##### Remarks.

Measurements of the specimens collected are similar to those of the original description, except for the width of dorsal shield (reported as 147 for the female holotype, probably a mistake). This is the first description of a male of this species.

#### Iphiseiodes setillus

Taxon classificationAnimaliaMesostigmataPhytoseiidae

Gondim Jr. & Moraes

Iphiseiodes setillus Gondim Jr. & Moraes, 2001: 75.
Iphiseiodes setillus : Moraes et al. 2004: 91.
Iphiseiodes setillus : Chant and McMurtry 2007: 98.

##### Specimens examined.

UESC, *Euterpe oleracea*, VII-2007 (1♀), *Theobroma cacao*, VII-2007 (1♀), I-2008 (2♀), IV-2008 (2♀); Fazenda Bela Vista, *Theobroma cacao*, XI-2007 (1♀); Fazenda Frupical, *Theobroma cacao*, VII-2007 (1♀).

##### Female.

Six specimens measured. Dorsal shield 312 (256–364) long and 224 (212–246) wide, *j1* 8 (6–8), *j3* 15 (14–16), *j4* 15 (14–15), *j5* 16, *j6* 17 (15–18), *J2* 17 (17–18), *J5* 9 (7–9), *z2* 11, *z4* 16 (15–17), *z5* 14 (12–15), *Z1* 17 (16–18), *Z4* 18 (16–19), *Z5* 25 (21–27), *s4* 15 (14–16), *S2* 17 (16–18), *S4* 17 (15–18), *S5* 16 (14–17), *r3* 11 (10–11), *R1* 11 (11–12); distances between *St1*–*St3* 41 (40–42), *St2–St2* 60 (58–61) and *St5–St5* 80 (76–84); ventrianal shield 77 (75–79) long, 95 (86–98) wide at level of *ZV2* and 75 (73–80) wide at level of anus; movable cheliceral digit 22 (21–23) long, with 2 teeth; fixed cheliceral digit 19 (18–20) long, with 6 teeth; calyx of spermatheca 10 (8–11) long; *Sge I* 8 (7–8), *Sge II* 11 (11–12), *Sge III* 13 (13–14), *Sti III* 14 (13–15), *Sge IV* 13 (13–14), *Sti IV* 14 (14–15), *St IV* 20 (19–20).

##### Remarks.

Measurements of specimens collected are similar to those of the original description. This is the first record of this species in Bahia.

#### Iphiseiodes zuluagai

Taxon classificationAnimaliaMesostigmataPhytoseiidae

Denmark & Muma

Iphiseiodes zuluagai Denmark & Muma, 1972: 23.
Iphiseiodes zuluagai : Moraes et al. 2004: 91.
Iphiseiodes zuluagai : Chant and McMurtry 2007: 98.

##### Specimens examined.

UESC, *Cocos nucifera*, VII-2007 (6♀), I-2008 (1♂), IV-2008 (1♀), *Carica papaya*, V-2007 (2♀), *Theobroma grandiflorum*, VIII-2007 (2♀), *Elaeis guineensis*, VII-2007 (1♀), I-2008 (1♀); CEPLAC, *Cocos nucifera*, fruit, XII-2008 (1♀); Fazenda Barra, *Anacardium occidentale*, VIII-2007 (1♀), *Annona muricata*, XI-2007 (1♀); Fazenda Bela Vista, *Theobroma cacao*, IV-2007 (3♀, 2♂), XI-2007 (2♀); Fazenda Formiga, *Annona muricata*, VIII-2007 (1♀), *Cocos nucifera*, VIII-2007 (1♀); Fazenda Liberdade, *Euterpe oleracea*, V-2007 (1♀), *Theobroma cacao*, V-2007 (3♀); Fazenda Monte Alegre, *Cocos nucifera*, I-2008 (2♀, 1♂), V-2008 (2♀, 2♂); Fazenda Nossa Senhora Auxiliadora, *Pouteria caimito*, IV-2007 (2♀); Fazenda Terra Nova, *Cocos nucifera*, IX-2007 (1♂); Sítio Agrotropical, *Cocos nucifera*, III-2007 (1♀, 1♂); Sítio Sabino, *Mangifera indica*, VIII-2007 (5♀).

##### Female.

Thirteen specimens measured. Dorsal shield 353 (307–397) long, 291 (256–333) wide, *j1* 21 (15–25), *j3* 29 (20–35), *j4* 2 (2–3), *j5* 3 (2–3), *j6* 3 (3–4), *J2* 3 (3–4), *J5* 4 (3–4), *z2* 3 (2–3), *z4* 3 (2–3), *z5* 3 (2–3), *Z1* 3 (2–4), *Z4* 4 (3–5), *Z5* 131 (105–144), *s4* 109 (87–122), *S2* 3 (3–4), *S4* 3 (2–4), *S5* 3 (2–4), *r3* 6 (4–7), *R1* 4 (4–5); distances between *St1*–*St3* 49 (41–53), *St2–St2* 77 (70–82) and *St5–St5* 104 (89–115); ventrianal shield 99 (82–110) long, 120 (96–133) wide at level of *ZV2* and 88 (70–112) wide at level of anus; movable cheliceral digit 36 (34–37) long; fixed cheliceral digit 29 (25–31) long; calyx of spermatheca 7 (5–8) long; *Sge I* 51 (40–60), *Sge II* 33 (26–40), *Sge III* 51 (39–59), *Sti III* 29 (23–32), *Sge IV* 93 (63–107), *Sti IV* 62 (48–72), *St IV* 38 (30–44).

##### Male.

Eight specimens measured. Dorsal shield 287 (269–312) long, 223 (205–256) wide, *j1* 21 (15–25), *j3* 37 (32–40), *j4* 2 (2–3), *j5* 3 (2–3), *j6* 3 (3–4), *J2* 4 (3–4), *J5* 4 (3–5), *z2* 3 (2–4), *z4* 3 (2–3), *z5* 3 (2–3), *Z1* 3(3–4), *Z4* 4 (3–6), *Z5* 93 (77–108), *s4* 82(64–92), *S2* 4 (3–4), *S4* 3 (2–5), *S5* 5 (3–7), *r3* 7 (6–8), *R1* 5 (3–5); ventrianal shield 110 (100–121) long and 169 (148–187) wide at anterior corners; shaft of spermatodactyl 16(15–18) long; *Sge I* 41 (31–47), *Sge II* 30 (24–33), *Sge III* 40 (29–46), *Sti III* 26 (20–33), *Sge IV* 65 (52–72), *Sti IV* 49 (38–56), *St IV* 35 (26–46).

##### Remarks.

Measurements of the specimens collected are similar to those of the original description, except for the longer *r3* and *R1* (2 for the holotype). The calyx of the spermatheca is shorter than reported by Lofego et al. (2009) for specimens from São Paulo state [14 (12–15)]. Measurements of male specimens fit the measurements of the allotype male and those of Lofego et al. (2004).

#### Paraamblyseius multicircularis

Taxon classificationAnimaliaMesostigmataPhytoseiidae

Gondim Jr. & Moraes

Paraamblyseius multicircularis Gondim Jr. & Moraes, 2001: 79.
Paraamblyseius multicircularis : Moraes et al. 2004: 158.
Paraamblyseius multicircularis : Chant and McMurtry 2007: 103.

##### Specimens examined.

UESC, *Euterpe oleracea*, I-2008 (1♀), *Theobroma cacao*, VII-2007 (3♀), I-2008 (1♀, 1♂), IV-2008 (2♀).

##### Female.

Seven specimens measured. Dorsal shield 331 (302–360) long, 248 (225–270) wide, *j1* 13 (13–15), *j3* 24 (22–25), *j4* 30 (27–33), *j5* 38 (34–42), *j6* 38 (35–42), *J2* 42 (38–44), *J5* 13 (12–14), *z2* 29 (27–31), *z4* 36 (33–41), *z5* 28 (24–30), *Z1* 45 (43–49), *Z4* 44 (40–46), *Z5* 29 (27–31), *s4* 42 (37–46), *S2* 33 (30–35), *S4* 33 (31–35), *S5* 24 (23–26), *r3* 13, *R1* 17 (16–18); distances between *St1*–*St3* 32 (30–34), *St2–St2* 64 (62–67) and *St5–St5* 100 (95–104); ventrianal shield 110 (103–117) long, 191 (182–201) wide at level of *ZV2* and 117 (110–125) wide at level of anus; movable cheliceral digit 20 (19–21) long, with 2 teeth; fixed cheliceral digit 20 (19–21) long; calyx of spermatheca 18 (17–20) long.

##### Male.

One specimen measured. Dorsal shield 218 long, 179 wide, *j1* 13, *j3* 23, *j4* 26, *j5* 30, *j6* 28, *J2* 30, *J5* 8, *z2* 18, *z4* 30, *z5* 21, *Z1* 37, *Z4* 38, *Z5* 23, *s4* 41, *S2* 31, *S4* 24, *S5* 18, *r3* 11, *R1* 12; ventrianal shield 92 long and 153 wide at anterior corners; shaft of spermatodactyl 20 long.

##### Remarks.

Measurements of the females collected are similar to those of the original description, except for longer dorsal shield (263 for the holotype). This is the first description of a male of this species and the first record of this genus in Bahia.

#### Paraphytoseius orientalis

Taxon classificationAnimaliaMesostigmataPhytoseiidae

(Narayanan, Kaur & Ghai)

Typhlodromus (Amblyseius) orientalis Narayanam et al., 1960: 394.
Paraphytoseius orientalis : Moraes et al. 2004: 162.
Paraphytoseius orientalis : Chant and McMurtry 2007: 53.

##### Specimens examined.

Fazenda Nossa Senhora Auxiliadora, *Psidium guajava*, XI-2007 (2♀).

##### Female.

Two specimens measured. Dorsal shield 270, 282 long, 159 wide, *j1* 31, 38, *j3* 80, 88, *j4* 4, 5, *j5* 3, 5, *j6* 6, *J5* 4, *z2* 8, 9, *z4* 8, *z5* 4, 5, *Z1* 6, 7, *Z4* 69, 76, *Z5* 106, 110, *s4* 123, 127, *r3* 40, 47, *R1* 25, 26; distances between *St1*–*St3* 64, *St2–St2* 64 and *St5–St5* 80; ventrianal shield 85, 107 long, 55 wide at level of *ZV2* and 51 wide at level of anus; movable cheliceral digit 29, 31 long, with 2 teeth; fixed cheliceral digit 24, 25 long, with 11 teeth; calyx of spermatheca 4, 5 long; *Sge IV* 26, 31, *Sti IV* 36, 37, *St IV* 43, 46.

##### Remarks.

Only measurements of the dorsal shield and of the longer setae were given in the original description. The specimens collected are slightly smaller and concurrently have slightly shorter setae than the holotype. Measurements of the specimens collected are similar to those of the original description of its junior synonym, *Paraphytoseius multidentatus* Swirski & Shechter, 1961, except for the longer *Z5* (76–91 in the latter). They also are similar to the measurements provided by Lofego et al. (2009).

#### Phytoscutus sexpilis

Taxon classificationAnimaliaMesostigmataPhytoseiidae

Muma

Phytoscutus sexpilis Muma, 1961: 275.
Phytoscutus sexpilis : Moraes et al. 2004: 166.
Phytoscutus sexpilis : Chant and McMurtry 2007: 101.

##### Specimens examined.

Fazenda Nossa Senhora Auxiliadora, *Psidium guajava*, VII-2007 (1♀).

##### Female.

One specimen measured. Dorsal shield 350 long, 330 wide, *j1* 15, *j3* 33, *j4* 12, *j6* 12, *J5* 9, *z2* 11, *z4* 14, *z5* 10, *Z1* 15, *Z4* 192, *Z5* 276, *s4* 184, *S4* 9, *S5* 9, *r3* 14, *R1* 16; distances between *St1*–*St3* 48, *St2–St2* 57 and *St5–St5* 87; ventrianal shield 137 long, 165 wide at level of *ZV2* and 128 wide at level of anus; movable cheliceral digit 25 long; fixed cheliceral digit 17 long; calyx of spermatheca 15 long; *Sge IV* 84, *Sti IV* 69.

##### Remarks.

Measurements of the specimen collected fit the redescription of the holotype given by Yoshida-Shaul and Chant (1997), except for the shorter *j1*, *J5*, *z4*, *Z1*, *S4* and *S5* and the longer *j4* and *r3* [respectively, 21, 13, 22, 26, 13, 14, 9 and 10]. Gondim Jr. and Moraes (2001) reported specimens from São Paulo state to have longer *z2*, *z4* and *S5* [respectively, 16, 26 and 12].

#### Proprioseiopsis ovatus

Taxon classificationAnimaliaMesostigmataPhytoseiidae

(Garman)

Amblyseiopsis ovatus Garman, 1958: 78.
Proprioseiopsis ovatus : Moraes et al. 2004: 184.
Proprioseiopsis ovatus : Chant and McMurtry 2007: 89.

##### Specimens examined.

UESC, *Musa sapientum*, IV-2008 (1♀); Fazenda Terra Nova, *Euterpe oleracea*, I-2008 (1♀); Sítio Sabino, *Passiflora edulis*, XI-2007 (1♀).

##### Female.

Three specimens measured. Dorsal shield 366 (360–372) long, 306 (287–320) wide, *j1* 31 (30–32), *j3* 69 (63–72), *j4* 7, *j5* 4 (3–5), *j6* 12 (10–13), *J5* 9, *z2* 42 (32–50), *z4* 22 (21–23), *z5* 8, *Z1* 22 (20–24), *Z4* 110 (101–115), *Z5* 84 (79–90), *s4* 102 (92–112), *S2* 22 (21–23), *S4* 12 (11–12), *S5* 11 (10–11), *r3* 20 (20–21), *R1* 11 (10–12); distances between *St1*–*St3* 58 (57–58), *St2–St2* 74 (72–76) and *St5–St5* 91 (90–92); ventrianal shield 114 (113–115) long, 111 (110–112) wide at level of *ZV2* and 91 (85–96) wide at level of anus; movable cheliceral digit 32 (30–34) long; fixed cheliceral digit 31 (30–32) long; calyx of spermatheca 15 (12–15) long; *Sge II* 18 (17–18), *Sge III* 30 (29–31), *Sti III* 26 (25–27), *Sge IV* 61 (60–62), *Sti IV* 41 (35–45), *St IV* 89 (88–90).

##### Remarks.

Measurements of the specimens collected fit the redescription of the holotype given by Moraes and McMurtry (1983), except for the shorter *S4* and longer width of dorsal shield, *J5* and *Sge IV* [respectively, 23, 252, 4 and 48 in the latter]. The measurements fit the redescription given by Lofego et al. (2009) and Ferla et al. (2011) for Brazilian specimens.

#### Proprioseiopsis dominigos

Taxon classificationAnimaliaMesostigmataPhytoseiidae

(El-Banhawy)

Amblyseius dominigos El-Banhawy, 1984: 130.
Proprioseiopsis dominigos : Moraes et al. 2004: 175.
Proprioseiopsis dominigos : Chant and McMurtry 2007: 89.

##### Specimens examined.

Fazenda Monte Alegre, *Musa sapientum*, IX-2007 (1♀), *Psidium guajava*, I-2008 (1♀, 1♂); Sítio Agrotropical, *Cocos nucifera*, III-2007 (1♀).

##### Female.

Three specimens measured. Dorsal shield 397 long, 307 wide, *j1* 35, *j3* 106 (100–114), *j4* 3, *j5* 3, *j6* 4 (3–4), *J5* 6 (5–7), *z2* 32 (31–33), *z4* 54 (50–57), *z5* 3, *Z1* 6 (5–7), *Z4* 128 (123–131), *Z5* 120 (114–125), *s4* 123 (118–127), *S2* 8 (6–10), *S4* 8 (7–9), *S5* 5 (5–6), *r3* 28 (27–30), *R1* 9 (8–9); distances between *St1*–*St3* 54 (52–56), *St2–St2* 74 (71–75) and *St5–St5* 130 (129–130); ventrianal shield 117 (110–122) long, 138 (127–145) wide at level of *ZV2* and 113 (110–117) wide at level of anus; movable cheliceral digit 45 long; fixed cheliceral digit 40 long; calyx of spermatheca 22 (20–25) long; *Sge I* 31, *Sge II* 25, *Sge III* 31 (30–32), *Sti III* 29 (28–30), *Sge IV* 54 (52–56), *Sti IV* 37 (36–37), *St IV* 51 (46–56).

##### Male.

One specimen measured. Dorsal shield 333 long, 230 wide, *j1* 30, *j3* 82, *j4* 2, *j5* 3, *j6* 3, *J5* 4, *z2* 28, *z4* 43, *z5* 3, *Z1* 5, *Z4* 97, *Z5* 88, *s4* 90, *S2* 9, *S4* 7, *S5* 6, *r3* 17, *R1* 8; ventrianal shield 148 long and 183 wide at anterior corners; shaft of spermatodactyl 19 long; *Sge I* 22, *Sge II* 20, *Sge III* 21, *Sti III* 23, *Sge IV* 40, *Sti IV* 27, *St IV* 44.

##### Remarks.

Measurements of the specimens collected are similar to those of the original description. They also agree with those of Moraes et al. (2013), except for the longer *z2*, *z4* and *S4* [respectively, 21, 37 and 5 in the latter]. This is the first record of this species in Bahia.

#### Proprioseiopsis neotropicus

Taxon classificationAnimaliaMesostigmataPhytoseiidae

(Ehara)

Amblyseius neotropicus Ehara, 1966: 133.
Proprioseiopsis neotropicus : Moraes et al. 2004: 183.
Proprioseiopsis neotropicus : Chant and McMurtry 2007: 89.

##### Specimens examined.

UESC, *Cocos nucifera*, I-2008 (1♀); Fazenda Barra, *Theobroma cacao*, XI-2007 (2♀); Fazenda Liberdade, *Theobroma cacao*, V-2007 (1♀); Fazenda Monte Alegre, *Musa sapientum*, I-2008 (1♀); Fazenda Terra Nova, *Diospyros kaki*, V-2007 (1♀).

##### Female.

Four specimens measured. Dorsal shield 424 (376–453) long, 331 (300–371) wide, *j1* 34 (31–36), *j3* 47 (46–48), *j4* 6 (4–7), *j5* 5 (5–6), *j6* 7 (6–8), *J5* 5 (5–6), *z2* 26 (22–29), *z4* 21 (17–25), *z5* 6 (5–7), *Z1* 8 (7–8), *Z4* 120 (112–130), *Z5* 106 (98–116), *s4* 119 (107–126), *S2* 8 (7–8), *S4* 8 (7–8), *S5* 8 (7–9), *r3* 24 (21–26), *R1* 13 (10–17); distances between *St1*–*St3* 66 (65–66), *St2–St2* 85 (82–88) and *St5–St5* 109 (103–117); ventrianal shield 114 (108–120) long, 115 (105–122) wide at level of *ZV2* and 94 (91–97) wide at level of anus; movable cheliceral digit 40 (38–43) long, with 3 teeth; fixed cheliceral digit 33 (32–35) long; calyx of spermatheca 22 (20–25) long; *Sge I* 34 (32–39), *Sge II* 35 (33–40), *Sge III* 36 (34–38), *Sti III* 32 (31–34), *Sge IV* 79 (72–82), *Sti IV* 57 (50–65), *St IV* 69 (65–73).

##### Remarks.

Measurements of the specimens collected are similar to those of the original description, except for the longer *j3* and *r3* (respectively 32 and 15 in the latter). They also agree with those of Lofego et al. (2004), except for the longer *z4* (13 in the latter). This is the first record of this species in Bahia.

#### Proprioseiopsis pentagonalis

Taxon classificationAnimaliaMesostigmataPhytoseiidae

(Moraes & Mesa)

Amblyseius pentagonalis Moraes et al., 1991: 127.
Proprioseiopsis pentagonalis : Moraes et al. 2004: 186.
Proprioseiopsis pentagonalis : Chant and McMurtry 2007: 89.

##### Specimens examined.

Fazenda Monte Alegre, *Theobroma cacao*, I-2008 (1♀).

##### Female.

One specimen measured. Dorsal shield 328 long, 207 wide, *j1* 24, *j3* 23, *j4* 6, *j5* 6, *j6* 7, *J5* 10, *z2* 10, *z4* 11, *z5* 6, *Z1* 9, *Z4* 102, *Z5* 164, *s4* 74, *S2* 10, *S4* 10, *S5* 8, *r3* 18, *R1* 11; distances between *St1*–*St3* 63, *St2–St2* 69 and *St5–St5* 61; ventrianal shield 108 long, 85 wide at level of *ZV2* and 71 wide at level of anus; movable cheliceral digit 35 long; fixed cheliceral digit 30 long; calyx of spermatheca 50 long; *Sge II* 25, *Sge III* 22, *Sti III* 17, *Sge IV* 58, *Sti IV* 37, *St IV* 52.

##### Remarks.

Measurements of the specimen collected are similar to those of the original description and of Moraes et al. (2013) for specimens from São Paulo state. This is the first record of this species in Bahia.

#### Typhlodromalus peregrinus

Taxon classificationAnimaliaMesostigmataPhytoseiidae

(Muma)

Typhlodromus peregrinus Muma, 1955: 270.
Typhlodromalus peregrinus : Moraes et al. 2004: 202.
Typhlodromalus peregrinus : Chant and McMurtry 2007: 111.

##### Specimens examined.

CEPLAC, *Cocos nucifera*, fruits, XII-2008 (1♀), XII-2009 (1♀); Fazenda Barra, *Theobroma cacao*, XI-2007 (1♀); Fazenda Bela Vista, *Psidium guajava*, IV-2007 (1♀), XI-2007 (1♀); Fazenda Kamuí, *Cocos nucifera*, XI-2007 (1♀); Fazenda Nossa Senhora Auxiliadora, *Carica papaya*, VII-2007 (1♀).

##### Female.

Five specimens measured. Dorsal shield 351 (335–399) long, 226 (192–302) wide, *j1* 28 (27–29), *j3* 33 (31–37), *j4* 14 (12–15), *j5* 14 (11–15), *j6* 18 (15–20), *J2* 18 (14–21), *J5* 9 (8–10), *z2* 20 (18–22), *z4* 28 (27–30), *z5* 14 (11–15), *Z1* 25 (21–27), *Z4* 46 (41–52), *Z5* 64 (58–67), *s4* 41 (37–44), *S2* 30 (26–36), *S4* 25 (19–27), *S5* 12 (9–14), *r3* 20 (18–21), *R1* 16 (15–17); distances between *St1*–*St3* 64 (62–65), *St2–St2* 61 (60–63) and *St5–St5* 72 (69–74); ventrianal shield 109 (102–114) long, 64 (62–66) wide at level of *ZV2* and 61 (59–63) wide at level of anus; movable cheliceral digit 33 (30–35) long, with 3 teeth; fixed cheliceral digit 28 (25–30) long; calyx of spermatheca 22 (15–24) long; *Sge I* 15 (10–18), *Sge II* 18 (15–21), *Sge III* 28 (25–30), *Sti III* 18 (16–19), *Sge IV* 44 (39–47), *Sti IV* 21 (17–29), *St IV* 62 (58–70).

##### Remarks.

Setal measurements were not given in the original description. Measurements of the specimens collected are similar to those of McMurtry (1983), except for the longer *Z1* (16-17 in the latter); they also agree with the redescription given by Moraes et al. (2013).

#### Typhlodromips mangleae

Taxon classificationAnimaliaMesostigmataPhytoseiidae

De Leon

Typhlodromips mangleae De Leon, 1967: 28.
Typhlodromips mangleae : Moraes et al. 2004: 217.
Typhlodromips mangleae : Chant and McMurtry 2007: 63.

##### Specimens examined.

Sítio São Jorge, *Cocos nucifera*, XI-2007 (1♀).

##### Female.

One specimen measured. Dorsal shield 343 long and 220 wide, *j1* 18, *j3* 20, *j4* 10, *j5* 10, *j6* 11, *J2* 12, *J5* 9, *z2* 11, *z4* 10, *z5* 10, *Z1* 12, *Z4* 36, *Z5* 73, *s4* 22, *S2* 12, *S4* 10, *S5* 8, *r3* 14, *R1* 14; distances between *St1*–*St3* 56, *St2–St2* 62 and *St5–St5* 63; ventrianal shield 115 long, 87 wide at level of *ZV2* and 85 wide at level of anus; movable cheliceral digit 27 long; fixed cheliceral digit 23 long; calyx of spermatheca 5 long; *Sge I* 27, *Sge II* 26, *Sge III* 31, *Sti III* 24, *Sge IV* 43, *Sti IV* 36, *St IV* 53.

##### Remarks.

Measurements of the specimens collected are similar to those of the original description, except for shorter calyx of spermatheca (9 in the holotype). Lofego et al. (2004, 2009) reported a slightly longer *Z4* [respectively, 39 (37–41) and 38 (35–45)]. Measurements of the specimens collected are similar to those of Gondim Jr. and Moraes (2001). This is the first record of this species in Bahia.

#### Typhlodromips theobromae

Taxon classificationAnimaliaMesostigmataPhytoseiidae

Souza, Oliveira & Gondim Jr.

Typhlodromips theobromae : Souza et al. 2010: 49.

##### Specimens examined.

UESC, *Theobroma cacao*, VII-2007, IV-2008 (3♀, 2♂); Fazenda Monte Alegre, *Theobroma cacao*, V-2007 (1♀), *Mangifera indica*, V-2008 (2♀, 3♂); Fazenda Terra Nova, *Theobroma cacao*, IX-2007, I-2008 (4♀).

##### Female.

Ten specimens measured. Dorsal shield 301 (288–312) long, 211 (194–246) wide; *j1* 19 (17–21), *j3* 23 (17–26), *j4* 8 (6–10), *j5* 8 (6–10), *j6* 10 (8–12), *J2* 10 (8–15), *J5* 7 (7–8), *z2* 10 (8–12), *z4* 18 (16–21), *z5* 7 (6–9), *Z1* 13 (8–17), *Z4* 45 (43–47), *Z5* 56 (46–60), *s4* 23 (20–25), *S2* 15 (11–19), *S4* 10 (6–16), *S5* 9 (6–11), *r3* 11 (10–12), *R1* 9 (7–10); distances between *St1*–*St3* 58 (55–60), *St2–St2* 72 (68–74) and *St5–St5* 65 (61–65); ventrianal shield 88 (82–93) long, 91 (86–93) wide at level of *ZV2* and 74 (70–79) wide at level of anus; movable cheliceral digit 39 (38–40) long, with 3 teeth; fixed cheliceral digit 30 long, with 15 teeth; calyx of spermatheca 9 (7–10) long; *Sge II* 14 (12–16), *Sge III* 15 (14–18), *Sti III* 14 (12–15), *Sge IV* 31 (27–35), *Sti IV* 15(12–18), *St IV* 28 (25–30).

##### Male.

Five specimens measured; dorsal shield 229 (212–241) long and 160 (152–176) wide; *j1* 16 (14–18), *j3* 22 (19–24), *j4* 9 (8–9), *j5* 8 (8–9), *j6* 10 (9–10), *J2* 8 (8–9), *J5* 6 (5–6), *z2* 10 (8–11), *z4* 19 (17–22), *z5* 7 (7–8), *Z1* 9 (8–10), *Z4* 33 (31–35), *Z5* 36 (33–39), *s4* 21 (20–22), *S2* 13 (12–14), *S4* 9 (8–10), *S5* 7 (6–7), *r3* 12 (11–12), *R1* 8 (7–10); ventrianal shield 91 (87–96) long and 132 (128–138) wide at anterior corners; shaft of spermatodactyl 15 long; *Sge I* 13 (12–14), *Sge II* 12 (12–13), *Sge III* 12 (11–13), *Sti III* 12 (11–13), *Sge IV* 19 (18–20), *Sti IV* 14 (12–16), *St IV* 24 (23–25).

##### Remarks.

Measurements of the specimens collected are similar to those of the original description.

### Phytoseiinae Berlese

#### Phytoseius latinus

Taxon classificationAnimaliaMesostigmataPhytoseiidae

El-Banhawy

Phytoseius latinus El-Banhawy, 1984: 141.
Phytoseius latinus : Moraes et al. 2004: 217.
Phytoseius latinus : Chant and McMurtry 2007: 129.

##### Specimens examined.

Fazenda Bela Vista, *Psidium guajava*, IV-2007 (7♀, 6♂), VII-2007 (4♀, 2♂), VIII-2007 (1♀), XI-2007 (1♀).

##### Female.

Five specimens measured. Dorsal shield 272 (256–302) long, 135 (128–148) wide; *j1* 19 (18–20), *j3* 32, *j4* 7 (7–8), *j5* 7, *j6* 9 (8–10), *J2* 9 (8–10), *J5* 7 (6–8), *z2* 10 (9–10), *z3* 37 (35–38), *z4* 11 (10–11), *z5* 7 (6–7), *Z4* 47 (45–50), *Z5* 52 (50–55), *s4* 51 (48–55), *s6* 59 (56–62), *r3* 36 (33–37), *R1* 14 (13–16); distances between *St1*–*St3* 62 (60–64), *St2–St2* 64 (63–65) and *St5–St5* 53 (52–55); ventrianal shield 90 (85–94) long, 51 (48–55) wide at level of *ZV2* and 45 (40–49) wide at level of anus; movable cheliceral digit 29 (28–30) long; fixed cheliceral digit 25 (24–26) long; calyx of spermatheca 15 (15–16) long; *Sge IV* 29 (27–30), *Sti IV* 28 (27–29), *St IV* 29 (27–30).

##### Male.

Two specimens measured. Dorsal shield 228, 236 long, 115, 123 wide, *j1* 15, 16, *j3* 28, 29, *j4* 7, 8, *j5* 6, 8, *j6* 7, 9, *J2* 7, *J5* 6, *z2* 8, 10, *z3* 31, 32, *z4* 9, 10, *z5* 6, 7, *Z4* 32, 33, *Z5* 27, 30, *s4* 37, 38, *s6* 43, 45, *r3* 28, 30, *R1* 10, 11; ventrianal shield 88 long and 126 wide at anterior corners; shaft of spermatodactyl 12, 14 long; *Sge IV* 17, 18, *Sti IV* 16, *St IV* 21, 25.

##### Remarks.

The females collected differ from the original description by having longer *j3* and *Z4* and shorter *j4*, *J5* and *z4* (respectively 24, 36, 10, 17 and 15 in the holotype). This is the first record of this species in Bahia.

#### Phytoseius woodburyi

Taxon classificationAnimaliaMesostigmataPhytoseiidae

De Leon

Phytoseius (Phytoseius) woodburyi De Leon, 1965: 130.
Phytoseius woodburyi : Moraes et al. 2004: 246.
Phytoseius woodburyi : Chant and McMurtry 2007: 131.

##### Specimens examined.

Fazenda Bela Vista, *Cocos nucifera*, XI-2007 (1♀), *Psidium guajava* VII-2007 (11♀).

##### Female.

Five specimens measured. Dorsal shield 279 (265–310) long, 145 (140–150) wide, *j1* 29 (28–31), *j3* 33 (33–35), *j4* 5 (5–6), *j5* 5 (5–6), *j6* 5 (5–6), *J5* 6 (5–8), *z2* 13 (13–15), *z3* 30 (28–33), *z4* 12 (10–13), *z5* 6 (5–8), *Z4* 87 (85–91), *Z5* 73 (68–78), *s4* 118 (110–125), *s6* 78 (73–82), *r3* 43 (40–46); distances between *St1*–*St3* 55 (53–58), *St2–St2* 58 (55–60) and *St5–St5* 58 (55–61); ventrianal shield 77 (65–99) long, 32 (28–45) wide at level of *ZV2* and 39 (35–47) wide at level of anus; movable cheliceral digit 21 (18–25) long; fixed cheliceral digit 21 (20–23) long; calyx of spermatheca 8 (6–10) long; *Sge IV* 8 (8–9), *Sti IV* 49 (46–52), *St IV* 27 (24–30).

##### Remarks.

Measurements of the specimens collected are similar to those of the original description, except for the longer *St IV* (20 in the holotype). Specimens collected in this study have the ventrianal shield longer than reported by Gondim Jr. and Moraes (2001) for specimens from São Paulo state [53 (50-58) in the latter]. This is the first record of this species in Bahia.

### Typhlodrominae Wainstein

#### Cocoseius elsalvador

Taxon classificationAnimaliaMesostigmataPhytoseiidae

Denmark & Andrews

Cocoseius elsalvador Denmark & Andrews, 1981: 155.
Cocoseius elsalvador : Moraes et al. 2004: 263.
Cocoseius elsalvador : Chant and McMurtry 2007: 132.

##### Specimens examined.

Fazenda Terra Nova, *Theobroma cacao*, IX-2007 (1♀), *Cocos nucifera*, IX-2007 (2♀); Sítio São Jorge, *Cocos nucifera*, XI-2007 (2♀).

##### Female.

Five specimens measured. Dorsal shield 302 (279–315) long, 166 (154–192) wide; *j1* 27 (25–28), *j3* 54 (51–56), *j4* 34 (32–37), *j5* 52 (50–55), *j6* 66 (62–70), *J2* 63 (58–67), *J5* 28 (22–31), *z2* 21 (20–21), *z3* 31 (30–32), *z4* 73 (68–77), *z5* 34 (29–37), *z6* 93 (88–98), *Z4* 74 (68–79), *Z5* 77 (73–81), *s4* 79 (77–82), *S4* 75 (69–80), *r3* 42 (37–46), *R1* 72 (67–75); distances between *St1*–*St3* 55 (54–56), *St2–St2* 60 (57–63) and *St5–St5* 59 (57–62); ventrianal shield 70 (63–75) long, 60 (50–64) wide at level of *ZV2* and 59 (55–63) wide at level of anus; movable cheliceral digit 26 (25–26) long; fixed cheliceral digit 25 (25–26) long; calyx of spermatheca 21 (20–21) long; *Sge IV* 49 (46–52), *Sti IV* 37 (34–40), *St IV* 76 (73–79).

##### Remarks.

Measurements of the specimens collected are similar to those of the original description, except for the longer *Sge IV* and *Sti IV* (respectively 39 and 25 in the holotype). Gondim Jr. and Moraes (2001) reported specimens from Pernambuco state to have longer *z2* [27 (25-29)].

#### Cocoseius palmarum

Taxon classificationAnimaliaMesostigmataPhytoseiidae

Gondim Jr., Moraes & McMurtry

Cocoseius palmarum Gondim Jr. et al., 2000: 1226.
Cocoseius palmarum : Moraes et al. 2004: 263.
Cocoseius palmarum : Chant and McMurtry 2007: 134.

##### Specimens examined.

UESC, *Cocos nucifera*, VII-2007 (3♀), I-2008 (1♀), IV-2008 (1♀); Fazenda Formiga, *Cocos nucifera*, VIII-2007 (1♀); Fazenda Terra Nova, *Cocos nucifera*, IX-2007 (1♀), I-2008 (2♀).

##### Female.

Eight specimens measured. Dorsal shield 270 (265–275) long, 182 (161–189) wide, *j1* 25 (23–27), *j3* 41 (38–45), *j4* 54 (52–59), *j5* 61 (57–64), *j6* 68 (65–70), *J2* 68 (65–72), *J5* 12 (11–13), *z2* 26 (21–28), *z3* 34 (31–36), *z4* 69 (65–75), *z5* 34 (31–38), *z6* 88 (85–92), *Z4* 73 (70–75), *Z5* 69 (67–71), *s4* 76 (72–81), *S4* 64 (60–68), *r3* 57 (52–65), *R1* 54 (50–59); distances between *St1*–*St3* 48 (46–50), *St2–St2* 55 (53–57) and *St5–St5* 63 (60–66); ventrianal shield 93 (88–94) long, 70 (67–73) wide at level of *ZV2* and 63 (60–65) wide at level of anus; movable cheliceral digit 25 (23–26) long, with 1 teeth; fixed cheliceral digit 24 (22–25) long, with 3 teeth; calyx of spermatheca 16 (15–18) long; *Sge IV* 37 (36–40), *Sti IV* 26 (25–28), *St IV* 53 (50–56).

##### Remarks.

Measurements of the specimens collected are similar to those of the original description, except for a slightly shorter calyx of spermatheca [21 (20–23) in the original description]. This is the first record of this species in Bahia.

#### Leonseius regularis

Taxon classificationAnimaliaMesostigmataPhytoseiidae

(De Leon)

Typhloseiopsis regularis De Leon, 1965: 123.
Leonseius regularis : Moraes et al. 2004: 275.
Leonseius regularis : Chant and McMurtry 2007: 161.

##### Specimens examined.

UESC, *Theobroma cacao*, VII-2007 (7♀), I-2008 (5♀), *Elaeis guineensis*, VII-2007 (1♂), *Artocarpus integrifolia*, VII-2007 (2♀); Fazenda Bela Vista, *Theobroma cacao*, IV-2007 (5♀, 1♂), XI-2007 (8♀), *Psidium guajava* VII-2007 (1♀), *Spondias mombin*, IV-2007 (1♀), *Cocos nucifera*, IV-2007 (1♀), XI-2007 (1♀), *Annona muricata*, IV-2007 (4♀), *Artocarpus integrifolia*, IV-2007 (1♀), *Genipa americana*, IV-2007 (3♀); Fazenda Monte Alegre, *Theobroma cacao*, V-2007 (6♀, 1♂), *Cocos nucifera*, IX-2007 (2♀); Fazenda Nossa Senhora Auxiliadora, *Theobroma cacao*, VII-2007 (2♀); Fazenda Terra Nova, *Theobroma cacao*, I-2008 (1♀, 1♂); Sítio Sabino, *Mangifera indica*, VIII-2007 (1♀), *Syzygium malaccense*, VIII-2007 (1♀).

##### Female.

Twelve specimens measured. Dorsal shield 366 (333–397) long, 257 (212–282) wide, *j1* 28 (25–30), *j3* 38 (35–42), *j4* 4 (3–5), *j5* 4 (3–5), *j6* 5 (5–6), *J2* 6 (5–8), *J5* 8 (7–9), *z2* 4 (3–5), *z3* 11 (9–14), *z4* 6 (5–7), *z5* 4 (3–5), *Z4* 101 (85–111), *Z5* 276 (262–292), *s4* 89 (71–98), *s6* 8 (6–12), *S5* 6 (5–7), *r3* 10 (8–12), *R1* 9 (7–11); distances between *St1*–*St3* 61 (59–66), *St2–St2* 70 (67–73) and *St5–St5* 73 (70–76); ventrianal shield 115 (98–128) long, 63 (55–70) wide at level of *ZV2* and 64 (55–70) wide at level of anus; movable cheliceral digit 32 (30–33) long, with 4 teeth; fixed cheliceral digit 28 (25–30) long; calyx of spermatheca 18 (17–18) long; *Sge I* 54 (50–57), *Sge II* 40 (37–43), *Sge III* 51 (47–55), *Sti III* 38 (33–44), *Sge IV* 119 (107–128), *Sti IV* 68 (55–82), *St IV* 54 (48–60).

##### Male.

Four specimens measured. Dorsal shield 258 (243–266) long, 171 (166–179) wide, *j1* 22 (20–24), *j3* 32 (30–34), *j4* 5 (4–6), *j5* 5 (4–6), *j6* 6 (5–7), *J2* 6 (4–8), *J5* 7 (6–8), *z2* 4 (3–5), *z3* 10 (8–12), *z4* 5 (4–7), *z5* 4 (3–5), *Z4* 63 (62–64), *Z5* 193 (185–202), *s4* 51 (48–55), *s6* 8 (6–10), *S5* 6 (5–8), *r3* 10 (8–11), *R1* 7 (7–8); ventrianal shield 111 (107–115) long and 141 (138–146) wide at anterior corners; shaft of spermatodactyl 18 (17–18) long; *Sge I* 36 (35–37), *Sge II* 30 (28–32), *Sge III* 38 (36–40), *Sti III* 29 (28–31), *Sge IV* 68 (66–70), *Sti IV* 42 (40–45), *St IV* 44 (42–48).

##### Remarks.

Measurements of the specimens collected are similar to those of the original description, except for the shorter *s4* (105 in the holotype). They also agree with those reported by Moraes et al. (2013). This is the first record of this genus in Bahia.

#### Metaseiulus ferlai

Taxon classificationAnimaliaMesostigmataPhytoseiidae

Moraes, McMurtry & Lopes

Metaseiulus (Metaseiulus) ferlai Moraes et al., 2006: 352.
Metaseiulus ferlai : Chant and McMurtry 2007: 174.

##### Specimens examined.

Fazenda Barra, *Anacardium occidentale*, VIII-2007 (1♀).

##### Female.

One specimen measured. Dorsal shield 350 long, 189 wide, *j1* 21, *j3* 21, *j4* 14, *j5* 14, *j6* 17, *J2* 20, *J5* 9, *z2* 17, *z3* 18, *z4* 20, *z5* 15, *Z4* 30, *Z5* 29, *s4* 21, *s6* 24, *S2* 24, *S5* 21, *r3* 21, *R1* 21; distances between *St1*–*St3* 63, *St2–St2* 66 and *St5–St5* 58; ventrianal shield 116 long, 87 wide at level of *ZV2* and 85 wide at level of anus; movable cheliceral digit 31 long; fixed cheliceral digit 29 long; calyx of spermatheca 25 long.

##### Remarks.

Measurements of the specimens collected fit the original description. This is the first record of this genus in Bahia.

#### Typhlodromina subtropica

Taxon classificationAnimaliaMesostigmataPhytoseiidae

Muma & Denmark

Typhlodromina subtropica Muma & Denmark, 1969: 412.
Typhlodromina subtropica : Moraes et al. 2004: 305.
Typhlodromina subtropica : Chant and McMurtry 2007: 169.

##### Specimens examined.

Fazenda Terra Nova, *Diospyros kaki*, V-2007 (1♀).

##### Female.

One specimen measured. Dorsal shield 353 long, 262 wide, *j1* 22, *j4* 35, *j5* 31, *J2* 46, *J5* 13, *z2* 32, *z3* 37, *z4* 43, *z5* 35, *Z4* 57, *Z5* 51, *s4* 43, *s6* 55, *S5* 54, *r3* 30, *R1* 21; distances between *St1*–*St3* 56, *St2–St2* 61 and *St5–St5* 77; ventrianal shield 125 long, 90 wide at level of *ZV2* and 72 wide at level of anus; movable cheliceral digit 28 long; fixed cheliceral digit 22 long; calyx of spermatheca 20 long.

##### Remarks.

Measurements of the specimens collected fit the redescription of the holotype given by Moraes and McMurtry (1983), except for the longer *Z5* (42 in the holotype).

#### Typhlodromus (Anthoseius) transvaalensis

Taxon classificationAnimaliaMesostigmataPhytoseiidae

(Nesbitt)

Kampimodramus transvaalensis Nesbitt, 1951: 55.
Typhlodromus (Anthoseius) transvaalensis : Moraes et al. 2004: 355.
Typhlodromus (Anthoseius) transvaalensis : Chant and McMurtry 2007: 169.

##### Specimens examined.

CEPLAC, *Cocos nucifera*, fruit, VII-2008 (1♀).

##### Female.

One specimen measured. Dorsal shield 369 long, 205 wide, *j1* 30, *j3* 38, *j5* 30, *J2* 42, *J5* 9, *z2* 22, *z3* 40, *z4* 42, *z5* 26, *Z4* 51, *Z5* 60, *s4* 44, *s6* 48, *S2* 52, *S4* 53, *S5* 9, *r3* 34, *R1* 37; distances between *St1*–*St3* 64, *St2–St2* 62 and *St5–St5* 71; ventrianal shield 120 long, 69 wide at level of *ZV2* and 66 wide at level of anus; movable cheliceral digit 30 long; fixed cheliceral digit 29 long; *Sge IV* 22, *Sti IV* 30, *St IV* 46.

##### Remarks.

Measurements of the specimen collected agree with the redescription of the holotype given by Schicha (1981), except the longer *j1* (23 in the holotype). They are similar to measurements given by Ferla et al. (2011), except for the shorter *j3* (48 in the latter).

### Key to the phytoseiid species collected in the present work (females)

**Table:** 

1	Setae *z3* and/ or *s6* present	**2**
–	Setae *z3* and *s6* absent	**3**
2	Setae *Z1*, *S2*, *S4* and *S5* absent; seta *r3* inserted on dorsal shield	**23**
–	At last one of the setae *Zl*, *S2*, *S4* or *S5* present; seta *r3* inserted on unsclerotised cuticle	**24**
3	Posterior margin of sternal shield lightly sclerotized, often indistinct, with a posteromedian projection	**4**
–	Posterior margin of sternal shield distinct, without a posteromedian projection	**5**
4	Ratio *s4*:*Z1*> 3; seta *Z4* not as long as distance between its base and base of *S4*; dorsal shield mostly smooth, with few anterolateral striae	***Amblydromalus manihoti* (Moraes)**
–	Ratio *s4*:*Z1*< 3; seta *Z4* longer than distance between its base and base of *S4*; dorsal shield reticulated	***Typhlodromalus peregrinus* (Muma)**
5	Setae *J2*, *S2*, *S4* and *S5* absent; lateral margin of dorsal shield with distinct incision at level of *s4*; setae *j3*, *s4*, *Z4* and *Z5* thickened, strongly serrate, inserted on tubercles	***Paraphytoseius orientalis* (Narayanan, Kaur & Ghai)**
–	Setae *J2* and *S2* present or absent, *S4* and *S5* present; lateral margin of dorsal shield without distinct incision at level of s4; setae *j3*, *s4*, *Z4* and *Z5* not markedly thickened, smooth to lightly serrate, not inserted on tubercles	**6**
6	Ratio *s4*:*Z1*< 4; body never red or dark brown; seta *J2* present	**7**
–	Ratio *s4*:*Z1*> 4 (except for *Iphiseiodes setillus* and *Paraamblyseius multicircularis*); some species heavily sclerotized, body red or dark brown; seta *J2* present or absent	**8**
7	Calyx of spermatheca dish-shaped; seta *Z4* not as long as distance between its base and base of *S5*; *Sge IV* sharp-tipped	***Typhlodromips mangleae* De Leon**
–	Calyx of spermatheca short-tubular; seta *Z4* at least as long as distance between its base and base of *S5*; *Sge IV* with tiny knob	***Typhlodromips theobromae* Souza, Oliveira & Gondim Jr.**
8	All shields generally strongly sclerotized; sternal shield usually wider than long; ventrianal shield usually as wide as long or wider than long; setae *z2* and/or *z4* rarely short/ minute	**9**
–	All shields lightly sclerotized; sternal shield about as wide as long; ventrianal shield longer than wide; setae *z2* and *z4* usually short/ minute	**18**
9	Seta *J2* present (if absent, then *j5* and *S2* also absent)	**10**
–	Seta *J2* absent; *j5* and *S2* present	**15**
10	Setae *j5*, *J2* and *S2* absent	***Phytoscutus sexpilis* Muma**
–	Setae *j5*, *J2* and *S2* present	**11**
11	Dorsal shield without marked circular ornamentation or reticulation; macrosetae present on legs I-IV	**12**
–	Dorsal shield with marked circular ornamentation or reticulate; macrosetae, if present, only on basitarsus IV	**14**
12	With one pair of enlarged metapodal plates; seta *Z4* longer than distance between its base and base of *Z5*	***Iphiseiodes metapodalis* (El-Banhawy)**
–	With 2 pairs of metapodal plates, none distinctly large; seta *Z4* shorter than distance between its base and base of *Z5*	**13**
13	Except for *j1*, *j3*, *s4* and *Z5*, dorsal shield setae short/ minute; setae *s4* and *Z5* considerably longer than others; setae *Z5*, *Sge IV* and *Sti IV* knobbed	***Iphiseiodes zuluagai* Denmark & Muma**
–	Dorsal shield setae of medium lengths, none considerably longer than others; setae *Z5*, *Sge IV* and *Sti IV* sharp-tipped	***Iphiseiodes setillus* Gondim Jr. & Moraes**
14	Dorsal, genital and ventrianal shields with circular ornamentation; dorsal shield setae of medium length, none considerably longer than others; seta *ZV3* absent; with a pair of enlarged metapodal plates; leg macrosetae absent	***Paraamblyseius multicircularis* Gondim Jr. & Moraes**
–	Dorsal, genital and ventrianal shields reticulate; some dorsal shield setae much longer than others; seta *ZV3* present; with 2 pairs of metapodal plates, none distinctly enlarged; *St IV* present	***Arrenoseius urquharti* (Yoshida-Shaul & Chant)**
15	Calyx of spermatheca tubular, flared near vesicle and inflate near atrium, longer than 40 μm	***Proprioseiopsis pentagonalis* (Moraes & Mesa)**
–	Calyx of spermatheca saccular or funnel-shaped, shorter than 30 μm	**16**
16	Seta *j3* at most 1.2 times longer than distance between their bases; calyx saccular	***Proprioseiopsis neotropicus* (Ehara)**
–	Seta *j3* at least 1.5 times longer than distance between their bases; calyx funnel-shaped	**17**
17	Seta *j3* longer than 100 μm; seta *Z5* longer than 110 μm; seta *z2* shorter than *z4*; calyx longer than 20 μm	***Proprioseiopsis dominigos* (El-Banhawy)**
–	Seta *j3* shorter than 75 μm; seta *Z5* shorter than 100 μm; seta *z2* longer than *z4*; calyx shorter than 20 μm	***Proprioseiopsis ovatus* (Garman)**
18	Ventral shield separated from anal shield	***Amblyseius perditus* Chant & Baker**
–	Ventral and anal shields fused, constituting a ventrianal shield	**19**
19	Calyx of spermatheca shorter than 13 μm; less than 4 times as long as width at median length	**20**
–	Calyx of spermatheca longer than 14 μm; over 5 times as long as width at median length	**22**
20	Seta *Z4* not as long as distance between its base and base of *Z5*; setae *s4* and *Z4* respectively 41–44 and 30–33 μm long; calyx cup-shaped, 6 μm long, increasing progressively in diameter toward the base	***Amblyseius impeltatus* Denmark & Muma**
–	Zeta *Z4* longer than distance between its base and base of *Z5*; setae *s4* and *Z4* respectively longer than 55 and 50 μm; calyx short-tubular, at least 8 μm long, somewhat constricted in the middle	**21**
21	Setae *j1*, *j3*, *s4*, *Z4* and *Z5* respectively 23–26, 35–39, 56–61, 60–66 and 130–155 µm long	***Amblyseius igarassuensis* Gondim Jr. & Moraes**
–	Setae *j1*, *j3*, s4, *Z4* and *Z5* respectively 33–42, 42–55, 91–120, 115–148 and 223–307 µm long	***Amblyseius operculatus* De Leon**
22	Setae *s4*, *Z4* e *Z5* respectively 105–111, 120–143 and 271–315 μm long; atrium nodular, distinct; major duct narrower than calyx	***Amblyseius aerialis* (Muma)**
–	Setae *s4*, *Z4* e *Z5* respectively 90–92, 100–115 and 227–246 μm long; atrium small, indistinct, incorporated into base of calyx; major duct approximately of the same diameter as calyx	***Amblyseius tamatavensis* Blommers**
23	Setae *J2* and *R1* present; seta *s4* shorter than *s6*; insertions of setae *Z4* and *Z5* distinctly separated; each macroseta of leg IV with a tiny knob, none markedly shorter than others	***Phytoseius latinus* El-Banhawy**
–	Setae *J2* and *R1* absent; seta *s4* longer than *s6*; insertions of setae *Z4* and *Z5* approximate; macrosetae on leg IV broadly clavate, *Sge IV* markedly shorter than others	***Phytoseius woodburyi* (De Leon)**
24	Seta *S5* absent; ventrianal shield with 1 or 3 pairs of pre-anal setae; dorsal shield setae not distally swollen	**25**
–	Setae *S5* present; ventrianal shield with 4 pairs of pre-anal setae (if with 3 pairs, then most dorsal shield setae distally swollen)	**26**
25	Dorsal shield lightly reticulate; ventrianal shield with one pair of preanal setae; seta *ZV3* present	***Cocoseius elsalvador* Denmark & Andrews**
–	Dorsal shield smooth, except for a few anterolateral striae; ventrianal shield with 3 pairs of preanal setae; seta *ZV3* absent	***Cocoseius palmarum* Gondim Jr., Moraes & McMurtry**
26	Seta *S4* present; ventrianal shield with 3 pairs of preanal setae	***Typhlodromus transvaalensis* (Nesbitt)**
–	Seta *S4* absent; ventrianal shield with 4 pairs of preanal setae	**27**
27	Dorsal setae, except *j1*, *j3*, *s4*, *Z4* and Z5, short/ minute; setae *s4*, *Z4* and *Z5* greatly longer than other dorsal setae; setae *JV4* and *ZV3* present	***Leonseius regularis* (De Leon)**
–	Pattern of dorsal setae lengths not as above; seta *JV4* absent; seta *ZV3* present or absent	**28**
28	Seta *S2* absent, seta *ZV3* present; seta *R1* inserted on unsclerotized cuticle; seta *s6* over twice as long as *R1*; seta *S5* longer than distance between its base and base of *Z5*	***Typhlodromina subtropica* Muma & Denmark**
–	Seta *S2* present, seta *ZV3* absent; seta *R1* on dorsal shield; seta *s6* less than twice as long as *R1*; seta *S5* not as long as distance between its base and base of *Z5*	***Metaseiulus ferlai* Moraes, McMurtry & Lopes**

## Discussion

Fifty-one phytoseiid species have been reported from Bahia (Bonato et al. 1999; Denmark and Muma 1973; Farias et al. 1981; Fiaboe et al. 2004, 2007; Lawson-Balagbo et al. 2008; Lofego et al. 2000, 2013; Moraes et al. 1993, 1994, 1997; Moraes and Denmark 1999; Moraes and McMurtry 1983; Noronha et al. 1997; Noronha and Moraes 1989; Oliveira et al. 2007; Souza et al. 2010, 2012). In the present study, fifteen species are reported for the first time in that state, raising the number of known species to sixty-six.

By far most of the species and of the specimens collected belong to Amblyseiinae (72 and 81%, respectively), followed by the Typhlodrominae (21 and 13%) and the Phytoseiinae (7 and 6%). Similar patterns were summarized by Castro and Moraes (2010) for similar surveys conducted in the Atlantic Forest of southeastern Brazil. There was a general trend for less specific phytoseiid species, i.e., those found on larger number of host plants, to be most abundant. Most of the phytoseiid species was found on a single or few host species. The largest numbers of phytoseiids on *Cocos nucifera*, *Theobroma cacao* and *Psidium guajava* suggest that the microhabitat on the leaves of these plants favor these predators, but should not be taken to indicate the preference of these mites for those plants, given that the collecting effort was not the same on all plant species. These higher numbers could be due to the fact that these plants were among the most common in the localities where the study was conducted.

The most diverse genus in the present study was *Amblyseius*, as also found by Lawson-Balagbo et al. (2008) on coconut palm in the coastal region of Bahia. *Amblyseius operculatus* was the most abundant species and the species found in the largest number of plants. In total, the total number of specimens of this species was higher than the sum of the second and third most common species, and they were found in every month of the year, except (probably by chance) in February.

A noticeable absence in this study was mites belonging to the genus *Euseius* Wainstein. Although species of this genus have been reported as diverse and numerous in surveys conducted on different crops in the inland semiarid region of Bahia (Moraes et al. 1993; Moraes and McMurtry 1983), they were not found in the present study nor in previous surveys conducted in the southern coastal region of Bahia (A.R. Oliveira, personal observation) on different plant species.

Total annual rainfall in the semiarid region in the inland of Bahia ranges between 700 and 1,300 mm (Moraes et al. 1993; Moraes and McMurtry 1983), whereas in southern coast it is approximately 1,700 mm, with no pronounced dry season (Almeida and Valle 2010). Daud and Feres (2005) reported significant correlations between the population levels of *Euseius citrifolius* Denmark & Muma and rainfall (negative) or pollen abundance (positive). Pollen is known to constitute an important part of the diet of *Euseius* species (McMurtry et al. 2013). Thus, the apparent absence (or scarcity) of *Euseius* species in the present work could be related to the high rainfall in the southern coastal region and low pollen availability in the tropical fruit trees plantations surveyed. Species of this genus were not rare in a similar survey conducted in the coast of São Paulo state (Castro and Moraes 2010), where rainfall is quite similar to that reported in the southern coastal region of Bahia (Climate-Data.org 2015). This apparent discrepancy could be related to the fact that in that study these species were only found on plants of spontaneous growth, which could be protected at a certain level from the direct effect of rainfall. Those were not sampled in the present study.

The results of this study may contribute to the determination of future research themes, to subsidize future implementation of the use of phytoseiids as biological control agents in the region where the study was conducted. A next step in this trajectory could involve studies under controlled laboratory conditions to evaluate the interactions between the most common predators found and the most common pest species.

## Supplementary Material

XML Treatment for Amblydromalus manihoti

XML Treatment for Amblyseius aerialis

XML Treatment for Amblyseius igarassuensis

XML Treatment for Amblyseius impeltatus

XML Treatment for Amblyseius operculatus

XML Treatment for Amblyseius perditus

XML Treatment for Amblyseius tamatavensis

XML Treatment for Arrenoseius urquharti

XML Treatment for Iphiseiodes metapodalis

XML Treatment for Iphiseiodes setillus

XML Treatment for Iphiseiodes zuluagai

XML Treatment for Paraamblyseius multicircularis

XML Treatment for Paraphytoseius orientalis

XML Treatment for Phytoscutus sexpilis

XML Treatment for Proprioseiopsis ovatus

XML Treatment for Proprioseiopsis dominigos

XML Treatment for Proprioseiopsis neotropicus

XML Treatment for Proprioseiopsis pentagonalis

XML Treatment for Typhlodromalus peregrinus

XML Treatment for Typhlodromips mangleae

XML Treatment for Typhlodromips theobromae

XML Treatment for Phytoseius latinus

XML Treatment for Phytoseius woodburyi

XML Treatment for Cocoseius elsalvador

XML Treatment for Cocoseius palmarum

XML Treatment for Leonseius regularis

XML Treatment for Metaseiulus ferlai

XML Treatment for Typhlodromina subtropica

XML Treatment for Typhlodromus (Anthoseius) transvaalensis
